# Surgical treatment of a twice recurrent chondrosarcoma of the pubic symphysis: a case report and review of the literature

**DOI:** 10.4076/1757-1626-2-6769

**Published:** 2009-06-29

**Authors:** George Petsatodis, Stavros I Stavridis, Dimitrios Karataglis, Anastasios Christodoulou

**Affiliations:** Orthopaedic Department of Aristotle University of Thessaloniki “G. Papanikolaou” General HospitalThessalonikiGreece

## Abstract

The case of a patient with a second recurrence of a chondrosarcoma of the pelvis and pubic symphysis is presented, in order to show the difficulties of the surgical treatment and the long course of the tumor.

A 56-year-old woman having already been operated upon twice within two decades, presented with a large, mass of the pubic symphysis, extending into the left proximal thigh.

Preoperative imaging revealed a large tumor occupying the pubic symphysis and the pubic bones up to the ischial tuberosities, extending into the soft tissues of the inner surface of the left thigh and displacing the urinary bladder, the urethra and the vagina.

Intraoperatively, a radical excision of the tumor was performed, including removal of the osseous substrate of the anterior pelvis. The anterior abdominal wall was supported with a special synthetic mesh secured on the osseous stumps in order to prevent visceral herniation. Histological examination showed grade I to II chondrosarcoma, while the patient’s postoperative course was uncomplicated.

At the latest follow-up two years postoperatively, the patient is pain-free and ambulatory with no signs of tumor recurrence, genitourinary complications or visceral herniation.

## Introduction

Chondrosarcoma is the third most common primary bone malignancy after myeloma and osteosarcoma, accounting for approximately 20% of bone sarcomas and mainly affecting the middle-aged population. It comprises a heterogeneous group of neoplasms that are characterized by cartilage matrix production from the tumor cells [1-3]. The pelvis is the commonest site of occurrence with the ilium being the most frequently involved bone, followed by the pubis and ischium [4]. Chondrosarcomas can either arise *de novo* as primary tumors, or less frequently, they can originate from previously existing benign cartilaginous tumors such as osteochondromas and enchondromas [2].

Although the majority of these tumors grow slowly, rarely metastasize and have a very good prognosis after surgery, local recurrence is a quite common occurrence especially following inadequate excision. Since radiotherapy and chemotherapy have proved ineffective in the treatment of chondrosarcomas, wide surgical excision remains the treatment of choice [1,5]. The complex anatomic relations of the pelvis, the close vicinity to vital structures, the lack of defined compartment borders and the risk of jeopardizing pelvic structural stability render effective surgical treatment of a pelvic chondrosarcoma a major challenge for the orthopaedic surgeon.

We hereby present a case of a twice recurrent pelvic chondrosarcoma occupying the pubic symphysis that was treated by wide marginal excision and abdominal wall reconstruction with the use of a special synthetic mesh in order to prevent a postoperative visceral herniation.

## Case presentation

**Figure 1.**
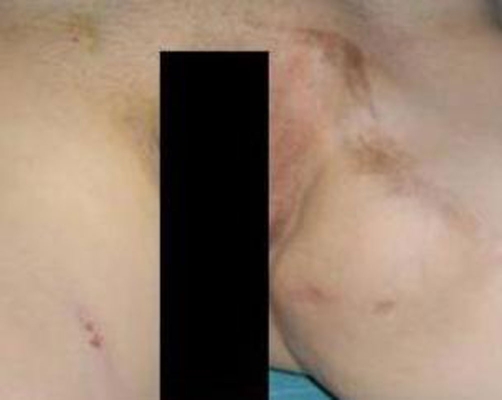
Clinical appearance of the tumor extending from the symphysis pubis to the left inner thigh.

A 56-year-old Greek woman presented with a large mass of the pubic symphysis, extending into the left hip (Figure 1). The patient reported an increase of the mass size within the last 6 months and otherwise minor complaints without significant pain. According to the patient’s history, 20 years ago she was subjected to a pelvic tumor removal followed by radiotherapy. 14 years postoperatively she was reoperated upon due to local tumor recurrence. The tumor had been partially removed, without bony resection, and the diagnosis of grade I chondrosarcoma was set.

**Figure 2.**
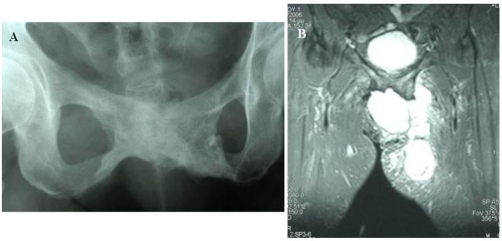
Preoperative plain X-ray **(A)** and MRI imaging of the tumor **(B)**.

Preoperative imaging revealed a large bilobular tumor occupying the pubic symphysis and the pubic bones up to the ischial tuberosities, extending into the soft tissues of the left thigh inner surface and displacing the urinary bladder, the urethra and the vagina (Figure 2A, 2B). No signs of invasion of adjacent organs, by the tumor were evident, while staging control did not reveal any remote metastases.

**Figure 3.**
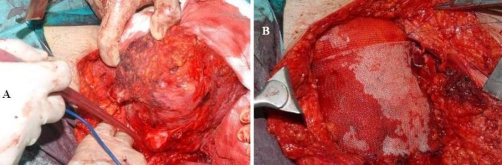
Intraoperative view of the operative field prior to the removal of the tumor **(A)** and following the mesh application **(B)**.

**Figure 4.**
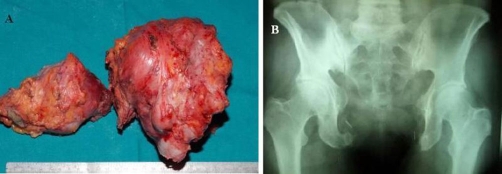
The removed chondrosarcoma **(A)** and a two-year-postoperative radiograph **(B)**.

The tumor was approached through a modified Pfannenstiel incision extending into to inner side of the left thigh. Intraoperatively, a radical excision of the tumor was performed (Figure 3A), including removal of the osseous substrate of the anterior pelvis, taking great care to ensure excision through unaffected tissues. In order to prevent a postoperative visceral herniation, the anterior abdominal wall was supported with a special synthetic mesh secured on the pelvic osseous stumps (Herniamesh® mesh, Herniamesh® S.r.l., Chivasso (TO), Italy) (Figure 3B). The removed tumor measured 16 cm in diameter (Figure 4A).

Histological examination confirmed a large pelvic chondrosarcoma of the pubic symphysis, being mainly Grade I and with small areas Grade II that destroyed the cortex and extended into the surrounding soft tissues. Excisional margins were found to be free of neoplasmatic cells.

The patient’s postoperative course was uncomplicated. Four months postoperatively she was able to ambulate full-weight-bearing. At the latest follow-up 2 years postoperatively, no urogenital complications or visceral herniation and no signs of relapse of the tumor were noted. The patient was symptom-free and satisfied with the results of her treatment.

## Discussion

Wide surgical tumor excision with adequate margins appears to be the procedure of choice in chondrosarcoma treatment, since this is the most effective way of reducing tumor recurrence rate [2-8]. Although local recurrence after inadequate surgical removal does not seem to significantly influence the overall survival, it must be kept in mind that each local recurrence bears the risk of the tumor evolving towards a higher-grade chondrosarcoma (up to 21.4%) [8]. Such transformation could consequently lead to an increased risk of metastases and subsequently result in a reduced overall survival rate [8,9]. A number of authors believe that the tumor grade is the only significant prognostic factor for survival and metastases [4,5].

A recent paper reported a recurrence rate of 100% in grade I pelvic chondrosarcoma following intralesional resection. Hence, although such a procedure may be considered a treatment option in some grade I chondrosarcomas of the long bones of the extremities, intralesional tumor resection should be avoided in the treatment of pelvic tumors [8]. The fact that in our case there was a tumor recurrence twice following partial removal supports the above findings.

Most chondrosarcomas of the pelvis are considerably larger than their appendicular counterparts, averaging 11 cm in size at the time of diagnosis. This could be partly attributed to the poor compartmentalization of the pelvis and the fact that most such tumors, as in our case, give raise to symptoms only after they have reached a relatively large size [10].

After inadequate initial excision there is increased likelihood of local recurrence because recurrent lesions have the propensity to implant in the pelvic soft tissues and do not remain as one isolated large lesion [9,10]. This seems to apply in our case where two recurrences of the tumor occurred within two decades.

The fact that in our case the first recurrence of the tumor took place more than a decade and the second almost 20 years post initial tumor resection indicates another aspect of the natural history of such tumors. Indeed, it is pointed out in the literature, that the index recurrence was seen as late as 87 months after the initial resection, while second recurrence occurred up to 12 years after index recurrent surgery. Thus, patients with a known recurrence should be followed for at least 15 to 20 years, a time considerably longer then that required for most other types of tumors. Unfortunately, even 10 years of monitored relapse-free status does not ensure cure of the disease [9]. Even in the cases of recurrent chondrosarcomas, the current literature suggests that every effort to achieve adequate resection margins should be made [9,10].

The choice of surgical procedure constitutes a factor of paramount importance. The surgeon has to maintain a subtle balance between ensuring adequate resection margins and the risk of endangering adjacent vital structures as well as the structural stability of the pelvis.

According to Enneking and Dunham [11] the pelvis is divided into 4 zones. The ilium is assigned number I, the periacetabular area number II, zone III corresponds to the pubis and ischium and zone IV to the sacrum. The extent of the excision needed according to the pelvic tumor location defines the reconstruction procedure required in order to reestablish stability and functionality of the pelvic ring. It is well documented in the literature that after zone III resection without impairment of the posterior structural elements a reconstruction of the anterior pelvic arch is not required, since pelvic stability is not affected [2]. Therefore, in our case we focused on achieving adequate resection margins through the bone but also through the soft tissues.

In order to reduce the possibility of a postoperative visceral herniation due to the removal of the anterior pelvic osseous substrate, we supported the anterior abdominal wall with a special synthetic mesh that was fixed upon the pelvic osseous stumps. This porous, monofilament polypropylene mesh acts as a "scaffold" enhancing growth of the patient's own tissue, which eventually incorporates the mesh into the surrounding area. This technique is often used in abdominal surgery for treatment of sizeable hernias.

## Conclusion

In conclusion, this case highlights the long natural course of recurrent pelvic chondrosarcoma and supports the current trend that wide resection should be the treatment of choice for such tumors even in the case of recurrence; while a limited reconstruction of the abdominal wall with a mesh may alleviate the consequences of the unavoidable bony resection.
